# Different Aspects of Informed Consent in Aesthetic Surgeries

**Published:** 2014-07

**Authors:** Nasrin Nejadsarvari, Ali Ebrahimi

**Affiliations:** 1 IMam Khomini Complex, Tehran University of Medical Sciences, Tehran, Iran;; 2 Department of Plastic Surgery, Trauma Research Center, Baqiyatallah University of Medical Sciences, Tehran, Iran

**Keywords:** Aesthetic, Surgery, Informed consent, Obligation, Patient

## Abstract

Providing an informed consent has an important role in promotion of medical treatments and reduction of judiciary litigations in this process. Today with cultural changes and wide propagation that is usually charming, the request for aesthetic surgery has an increasing trend. These problems with complexity of cosmetic surgeries lead to deeper differences of information between plastic surgeons and patients, so the discussion on giving information to a patient is of great importance. Regarding the elective choice of aesthetic surgeries, there is a need on providing a standard informed consent form. There are some problems on advertisements of aesthetic surgeries by non-plastic surgeons, taking insufficient or incorrect information to the patients affecting the patients’ autonomy. In fact, correct operative information should be share with the patients. Probable complications and alternative procedures should be presented to the patient to choose an operative option freely and without any charming. Obtaining a written informed consent can protect researchers and their sponsor institutions from any litigation. Patients with psychiatric problems can not benefit from aesthetic surgery and also they have no competency for giving any informed consent. So psychiatric problems can even worsen the surgical interventions. In this article, fundamentals of plastic surgery to provide an informed consent were reviewed and the legal and ethical considerations were evaluated.

## INTRODUCTION

Nowadays the patient’s autonomy has replaced the paternalism. Virtually, all prominent medical and research codes and institutional rules of ethics now hold that physicians and investigators must obtain the informed consent of patients prior to any substantial intervention.^1^ Patients have the right to choose to participate with their physicians in their own health care decision making.^2^ The United States of America reports on this topic were in fact the first approaches in USA at the beginning of the 18th century.^3^ It is necessary to take informed consent before any surgical intervention and surgeons must explain operative information to the patients. The duty to inform seeks to protect the patient interest in self decision making. Such an action would be more akin to an action in battery.^3^

These days, the popularity of cosmetic surgery is growing rapidly.^4^ The explosion in popularity can be attributed to several factors. The evaluation of safer, minimally invasive procedures, increased mass media attention and the great willingness of individuals to undergo cosmetic procedures are the means to enhance the physical appearance.^5^

Aesthetic surgery has evolved in the past years from a genuine medical process to a mere commodity.^6^ In Iran, some aesthetic surgeries have changed from a special medical topic to a job for financial profit for some physicians especially for non-plastic surgeons who have no special and academic trainings in this regard and unfortunately the media and newspapers may also help to these illegal actions. An aesthetic surgery that understands itself as part of a market would be nothing else than a part of a beauty industry which has the only aim to sell something but not the aim to help people.^6^ Most malpractice claims in cosmetic plastic surgeries are not a consequence of technical faults but because of inadequate selection criteria of the patients and lack of adequate communication between the patient and the surgeon.^7^ A written informed consent form remains an integral part of the communication between physicians and patients and importantly can facilitate the professional protection.^6^

Here, we evaluated the informed consent details, also the necessity of careful taking and conduct for a cosmetic surgery. The informed consent form is a very decisive part in aesthetic plastic surgery. As there is often no medical indication in a plastic surgery, the patient has to be informed about all facts of the operation, especially about its possible risk factors.^8^^,^^9^

## DEFINITIONS

The word of consent means to give an approval, assent or permission, and a voluntary agreement to another proposition.^10^^,^^11^ The consent of a patient for a surgical or medical procedure means the participation of the patient in a clinical intervention after achieving an understanding of the relevant medical facts and the risks involved.^12^

The word of consent by law means the voluntary agreement with an action proposed by another side. Consent is an act of reason that the person who provides the consent is of sufficient mental capacity and is in possession of all essential information in order to give a valid consent form. A person who is an infant is mentally incompetent, or a person who is under the influence of drugs is not capable of giving consent. Consent must also be free of coercion or fraud too.^13^

Providing an informed consent from a patient or the other recipient of services is based upon the principles of autonomy and privacy; this has become the requirement at the center of morally valid decision making in health care and research. Seven criteria were defined for an informed consent: (i) competence to understand and to decide, (ii) voluntary decision making, (iii) disclosure of material information, (iv) recommendation of a plan, (v) comprehension of terms and (vi) decision in favor of a plan, and (vii) authorization of the plan. A person gives informed consent only if all of these criteria are met. If all the criteria are met except that the person rejects the plan, that person makes an informed refusal.^13^

In non-emergency situations, a written informed consent is generally required before many medical procedures, such as surgery, including biopsies, endoscopy, and radiographic procedures involving catheterization. The physician must explain to the patient the diagnosis, the nature of the procedure, including the risks involved and the chances of success, and the alternative methods of treatment that are available. Nurses or other members on the health care team may be involved in filling out the consent form and witnessing the signature of the patient or the parent or guardian, if the patient is a minor. In medical research, the patient must be informed that the procedure is experimental, and that consent can be withdrawn at any time. In addition, the person signing the consent form must be informed regarding the risks and benefits of the experimental procedure and of alternative treatments.^13^

## RESULTS

Aesthetic plastic surgeon does not deal with a patient, but also deals with a healthy person that has exaggerated wishes and artificial reasons due to psychiatric gains. Some volunteers for cosmetic surgeries are due to emotional psychiatric gains; therefore, aesthetic surgeons must interview carefully and consider psychiatric disorders, including body dysmorphic syndrome, mood disorder, personality disorders, etc.^14^

Patients with these problems not only benefit from aesthetic surgery, but also they have no competency for giving informed consent. Furthermore, psychiatric problems may worsen after surgery; therefore, we must request psychiatric consultation for such patients. Plastic surgeons must learn about psychiatric disorders that may involve plastic surgery fields.^14^

Informed consent is an important prerequisite for plastic surgery; this action has a critical role in reducing legal claims. So, plastic surgeons must standardized informed consent according to any country law; also they need the ethical workshop for more training of young surgeons about legal and ethical problems, another problem about cosmetic surgery is doing these procedures by non-plastic surgeons that must be reduced with taking information to general populations. Computer automated informed consent will improve the efficiency to the consent process if these actions not damage physician- patient relationships.^14^

## DISCUSSION

Aesthetic surgeries were done electively, so the process of taking informed consent begins before office visiting of patients. The patient’s conception is dependent to media advertisement, and this causes patient’s exaggerated expectation from aesthetic surgery; therefore, legal claims in this type of surgeries are higher than other operations, especially if we do not explain treatment process and outcome in the patients. In cosmetic surgeries, taking informed consent is respect of patient’s autonomy. The reason for consent is information and understanding of a patient about treatment modalities and finally choosing one of the several options for treatment. Unfortunately, these days’ surgeons neglect preconditions and inform incomplete data to patients and taken consent is not informed consent of the patient.^14^

*Condition of Legal Consent*

Importantly, the legal duty as a doctor has been held to extend to accurately imparting information but not to ensure that it has been understood, this should be distinguished from any professional obligation. We have two aspects to understand a surgical option. First is understanding of the actual nature and purpose of the treatment. The second aspect denoted to the reasons of undertaking an aesthetic surgery. The patients would need to understand the risks, benefits and costs of having the procedure versus the same calculation for not having the procedure. Information can be less than is required, misleading or incorrect. The patient must be free without any obligation for consent, but more important is competence of the person to make such decisions. The most important legal factor for a person that takes informed consent is competency.^14^

A competent patient is a patient who is legally capable of giving the consent. Once the patients have established that they have legal competence, they can refuse consent to treatment for any rational or irrational reasons or for no reason at all, even if they lose their lives or suffer from significant harms as a result of this choice.^14^

An incompetent patient is a patient who is not able to give consent (for example, young children, some mentally-disabled and unconscious patients). For these patients, consent can be given by others (for example, those with parental responsibility, lasting powers of attorney or the court). In this context, consent is not clearly an expression of patient autonomy; rather, it amounts to a legal justification for the touching that is medically justified. So it is constrained by the best interests of the patient.^14^

Who are eligible to give consent to their treatment? Minors cannot make their individual inpatient mental health treatment decisions; this is the responsibility of their parent or guardian. Only adults who are consistently able to make well reasoned and would know decisions about their own mental health or medical care can give consent or revoke consent to their own treatment.^15^

*Elements of Ethical Consent*

Regarding the natural elective aesthetic surgeries, we need a standard informed consent form according to every country cultural and religious patterns. Elements of informed consents include threshold elements or precondition, and information consent. Threshold element has two parts: competence and voluntariness, according to these elements, the patient has self-determination right and can decide with autonomous in relation with his body without any other coercion.^1^

About incompetence persons, the guardian must decide and give informed consent to treatment measure. The elements of consent are disclosure, recommendation and its understanding.^1^ Informed consent is a process requiring a competent doctor, adequate transfer of informed and consent of the patient. It is not just a signature on a piece of paper.^16^ The surgeons or residents before any aesthetic surgery must take patients informed consent, furthermore, elements of consent forms must be revised and updated according to aesthetic surgical type.^16^

*History of Surgical Consent*

In medicine, doctors look for a document aiming at releasing them from any future responsibility to the patient or his family when any adverse event happens in follow up therapy. In 1767 in a leg fracture case, informed consent was taken.^17^ Thereafter, informed consent was revised and completed according to different kinds of anesthesia procedures. A patient should view it as a person who has the right of bodily self-determination.^18^

After the Second World War, there was a story on public reactions to the cruelties committed by Naji’s concentration camp doctors who performed horrible tests on patients without any prior information to the person or his approval. A code was written as a direct result of the Neurenberg trial that was an important step toward the development of the informed consent process in trials.^16^

The information that doctors legally require to give patients before their investigation or treatment has debated for over a decade. The informed patient is likely to be more cooperative and compliant and recover more quickly.^19^

*Problem Expression*

This public interest can limit in extreme situations the autonomous power of a competent person to give consent for a physical injury. So, consent is not a defense to a murder nor is a defense against a charge of actual bodily harm. The consent is an expression for the interest and is a valid self-determination, so the person giving consent must be aware of the choices he may face. They must have some grasp of the paths available, and the risks/benefits associated with each available path.^14^

There is elegance to a legal structure that requires doctors to owe a single comprehensive duty in negligence covering diagnosis and treatment, and the associated obligations to inform. Diagnosis and treatment are essentially the exercise of the medical professional skills and therefore, fall fairy into the arms of negligence.^14^

Aesthetic surgeries were done electively, so the process of taking informed consent begins before office visiting of patients. The patient’s conception is dependent on media advertisement, and this causes patient’s exaggerated expectation from aesthetic surgery. Therefore, legal claims in this type of surgeries are higher than other operations, especially if we do not explain the treatment process and outcome in the patients.^14^

In cosmetic surgeries, taking an informed consent is based upon the patient’s autonomy. The reason for consent is information and understanding of a patient about treatment modalities and finally choosing one of the several options for treatment. Unfortunately, these days, surgeons neglect preconditions and inform incomplete data for patients and taking consent is not an informed consent.^14^

Misinformation in relation to risk/benefit does not necessarily make the consent invalid and so a defense to an action for battery can be maintained. However, this does not preclude an action for negligence where the misinformation amounts to a breach of the duty to give sufficient information for a patient to reach a conclusion about whether they wish to accept the treatment or not. The fact that legal action for inadvertent misinformation in relation to the inherent risks/benefits of treatment lies in negligence rather than in battery leaves a legal structure that has some tension within it.^14^

A simple method for taking informed consent is to teach informed consent to surgical residents.^20^ However, teaching physicians for skill education of informed consent has not been very successful.^20^ There is the difference between countries about giving information to surgical patients before surgery. Today, oral information is temporary and fewer understanding for patients, especially in older ages or low IQ Patients with psychosomatic problems. Therefore, it is preferable to inform patients with written papers that have permanent staying and of course is applicable for legal claims.^14^

Plastic surgeons, preferably most times, spend their time in operation rooms; therefore, it is better than modern technology to be used for taking informed consent. Use of multimedia based on programs has been positively evaluated by patients and is significantly related to their disease and treatment measures. So it is valuable for an informed consent process.^21^

One of the most common causes of malpractice after complications and failure to make a timely diagnosis of an illness is a poor informed consent. The automated consent can improve the efficiency of the office and consent process.^22^

The rate of claims in aesthetic surgery is a number of times relative to other medical claims. This problem has some reasons. First of all, it is an incomplete supervision of physicians and the second reason is the wide range of aesthetic procedures. The third reason that is legally more important is the intervention of non-plastic surgeons or non-aesthetic surgeons. The goals of these non-qualified persons are giving more financial profit. However, they have no certification in plastic surgery and any duty or institutional authority on the problem which is very critical. Another problem is that the general population do not have sufficient information about qualified plastic surgeons or some physicians’ advertisement as the plastic surgeon.^22^

Another reason is the patient’s expectations from cosmetic surgeons as indeed, every person that is a volunteer for these surgeries is not a patient but also is a healthy person that wants to become more beautiful, and younger. Finally, an important reason for a claim in plastic surgeries is the psychosocial problem, including body dysmorphic diseases (BDD). Patients with BDD often have little or no insight on their illness, and some are, frankly, delusional convinced and their imaginary defect is real.^23^

The imaginary body defects are focused mostly on the face but may also be focused on the other body parts. These patients may have excessive preoccupations with their imaginary physical defects at the level of delusional or psychotic thinking that some of them may have suicidal ideas or may have attempted suicides during the past.^24^

There are strong evidences that patients with BDD do not benefit from cosmetic surgery, so it is enormously important to detect signs of BDD and refer them to the psychiatrist for consultation the surgeon may comfort great resistance in suggesting psychiatric consultation. The surgeons should not perform the requested surgery for the patient. Some of these patients may introduce a lawsuit or become violent toward the plastic surgeon. 

Diagnoses of these psychiatric disorders are not simple, and we must be careful in the office interview, especially when patients with multiple previous aesthetic surgeries have come to our office. Nevertheless, he or she is not satisfied with past surgeries. Patients with these problems not only benefit from aesthetic surgery, but also they have no competency for giving informed consent. Furthermore, psychiatric problems may be worsening after surgery. In the practice of cosmetic surgery, the standard for the duty of informed consent is crucial. Particularly, cosmetic surgery is generally elective; a malpractice claim is typically triggered when a patient is dissatisfied with the result. Unfortunately, for the cosmetic surgeon, it is difficult for a patient to comprehend that a meritorious malpractice claim is not equivalent to the unavoidable risks of a contemplated cosmetic procedure that result in the patient’s disappointment with the outcome.^25^

Generally, informed consent requires that the patients to be informed on risks of treatment, the prognosis, and alternative treatments before consenting to treatment. In a medical-malpractice case, the plaintiff bears the border of proving all the following: (i) The applicable standard of care, (ii) Breach of that standard by the defendant, (iii) Injury, and (iv) Proximate causation between the alleged breach and the injury. This standard is also applicable to an informed consent claim.^26^

Informed consent is provided by competent adult patients to ensure that their right to self-determination is respected. However, this doctrine also applies to others who do not possess competence, through their legal decision maker. Obtaining written informed consent can protect researchers and their sponsoring institutions from litigation.^26^

Fraud as to nature or purpose behind the act is enough to negate any apparent consent. Fraud as to the identity of the actor is also adequate to negate consent. It seems that what the patient consent is the performance of a medical act by a medically qualified practitioner.^26^ So fraud is induced qualifications for the patient to accept the treatment measure without familiarity with the nature or purpose of the undertaken actions nor to the identity of the actor. 

## CONFLICT OF INTEREST

The authors declare no conflict of interest. 

## Footenote

Please cite this paper as:

Nejadsarvari N, Ebrahimi A. Different Aspects of Informed Consent in Aesthetic Surgeries. World J Plast Surg 2014;3(2):81-86.
